# Grading Gliomas Capability: Comparison between Visual Assessment and Apparent Diffusion Coefficient (ADC) Value Measurement on Diffusion-Weighted Imaging (DWI)

**DOI:** 10.31557/APJCP.2020.21.2.385

**Published:** 2020

**Authors:** Warinthorn Phuttharak, Jureerat Thammaroj, Sakda Wara-Asawapati, Kobporn Panpeng

**Affiliations:** 1 *Department of Radiology, *; 2 *Department of Pathology, Faculty of Medicine, Khon Kaen University, Khon Kaen, Thailand. *

**Keywords:** Glioma, diffusion magnetic resonance imaging

## Abstract

**Background:**

To compare diagnostic accuracy between DWI visual scale assessment and ADC value measurement of solid portion of the tumor in grading gliomas.

**Methods:**

This retrospective study included 38 patients who had pathologically proven gliomas between January 2013 and August 2018 with 18 low grade and 20 high grade tumors. All patients underwent MRI and biopsy. Two readers reviewed DWI visual scale independently. Disagreement was resolved by consensus. One reviewer measured ADC value of entire solid part of the tumor in single axial slice with greatest dimension of tumor which was chosen by consensus. Two data sets of visual scale and ADC value were analyzed and comparison of diagnostic accuracy in glioma grading was done by using area under the curve (AUC) of receiver operating characteristic curve (ROC).

**Results:**

Visual scale and ADC value could be used to distinguish between low and high grade gliomas with a statistically significant difference. (P-value 0.002 and <0.001). Almost all high grade gliomas had visual scale 5. The sensitivity, specificity, PPV NPV and accuracy were 50%, 100%, 100% , 64.3%,73.68% respectively. The cutoff level for the ADC value was determined to be 1119.48 x10^-6^ mm^2^/s in differentiation between low and high grade gliomas with the sensitivity, specificity, PPV, NPV, accuracy of 90%, 88.89% , 90%, 88.9% and 89.47% respectively. There was no statistically significant difference(P-value = 0.163).

**Conclusion:**

Both Visual scale and ADC value were capable of differentiating between low and high grade gliomas. Although visual scale may not replace ADC measurement, larger scale prospective study is needed for validate this initial result.

## Introduction

Gliomas are the most common primary intracranial tumor. Although relatively rare, they cause significant mortality and morbidity. Glioblastoma, the most common glioma histology, has much shorter median survival time with much poorer survival than low grade glioma (McCormack et al., 1992; Ostrom and Barnholtz-Sloan, 2011). Accurate grading of gliomas is crucial for guiding the therapy and estimating patient prognosis. 

Diffusion-weighted imaging (DWI) is based on the irregular diffusion motion of water molecules and provides more detailed information at the cellular level than conventional magnetic resonance imaging (MRI). Because it is non-invasive and relatively low cost, DWI has been widely applied to the diagnosis of various diseases, including the detection of acute cerebral infarction, abscesses from cystic tumors and distinguishing epidermoid from arachnoid cysts (Schaefer et al., 2000; Stadnik et al., 2001; Holdsworth and Bammer, 2008). DWI can be integrated easily into a conventional MR examination at any time. DWI has also been explored as a rapid method for grading of brain tumors (Sugahara et al., 1999; Kono et al., 2001) either by visual evaluation of signal characteristics (Lam et al., 2002; Rollin et al., 2006) or quantitative analysis of apparent diffusion coefficient (ADC) values (Kan et al., 2006; Lee et al., 2008; Seo et al., 2008; Murakami et al., 2009; Jaremko et al., 2010).

Previous studies based primarily on quantitative ADC measurement in different ROI determination methods (Sugahara et al., 1999; Okamoto et al., 2000; Castillo et al., 2001; Kono et al., 2001; Stadnik et al., 2001; Lam et al., 2002; Higano et al., 2006; Kan et al., 2006; Lee et al., 2008; Seo et al., 2008; Murakami et al., 2009; Chen et al., 2010 ; Jaremko et al., 2010; Han et al., 2017; Van et al., 2018; Wang et al., 2018). To our knowledge, There are very few studies evaluating qualitative ADC visual score in glioma grading and no specifically comparison between ADC visual scoring and ADC measurement.The aim of the this study was to assess the value of DWI qualitative visual scale and DWI quantitative single slice ADC value measurement of solid portion of tumor in glioma grading and comparison diagnostic accuracy between both methods.

## Materials and Methods

*Study population*

This study was a retrospective study and conducted at Department of Radiology, Srinagarind Hospital, Faculty of Medicine, Khon Kaen University, Thailand. The inclusion criteria were patients with with pathologically glioma who had performed a 3T MRI and DWI/ADC prior to any treatment. The study period was between January 2013 and August 2018. The study protocol was approved by the ethic committee in human research, Khon Kaen University. Informed consents were not required by the approval of the ethic committee. 

*Imaging technique*

Using a routine brain protocol (Sagittal T1W imaging, Axial T1W and T2W and FLAIR imaging, Coronal T2W gradient imaging and post contrast axial, coronal and sagittal imaging); T1W images (550/10; number of signal acquired, 1; section thickness, 5 mm; intersection gap, 2 mm; matrix, 228 x 227;field of view[FOV], 25 x 25 cm), T2W images (5,000/92; number of signal acquired, 1; section thickness, 5 mm; intersection gap, 2 mm; matrix, 228 x 227; FOV, 25 x 25 cm). T1-weighted fat-suppressed gradient echo sequences after administration of gadolinium contrast 0.1 mmol/kg (Gadobutrol, Gadovist; Bayer Healthcare Pharmaceuticals) was also performed as part of the routine protocol.

All patients underwent DWI/ADC with a 3T MR scanner.(Phillips Achieva; Philips, Best, the Netherlands. A single shot echo-planar diffusion-weighted imaging sequence was performed. Imaging parameters of DWI were as followings: 1,819-8,000/85-93 (TR/TE) with diffusion sensitivities b=0 and b=1,000 s/mm^2^ for both scanners. The diffusion gradients were applied sequentially in three orthogonal directions to generate 2 sets of axial DW images. The ADC maps were automatically generated from the datasets of DWI images using the operating console and ADC were calculated.

*Post-Processing*

DWI data were transferred to a Synapse 3D workstation (Fujifilm Medical Systems, USA, Inc.) and ADC maps were generated.

*Imaging analysis*

*Visual scale assessment*

Two experienced neuroradiologists, blinded to patient history, clinical and pathological informations, independently reviewed the DWI and scored the signal intensity (SI) of tumors on a 1- to 5-point scales.

Qualitative visual scale was categorized into 5 point scales by comparison with SI of normal structure of the brain, we adapted from Seo et al., (2008) study which scored the SI of tumors on a 1- to 5-point scales. The 5 point scales assigned to tumor signal intensity on DWI obtained at b=1,000 s/mm^2^ was as followings: 1= markedly hypointense SI nearly equal to that of normal CSF; 2 = hypointense SI between those of normal CSF and normal subcortical white matter; 3 = isointense SI equal or similar to that of normal subcortical white matter; 4 = hyperintense SI between those of normal subcortical white matter and normal cortex; 5 = markedly hyperintense SI higher than that of normal cortex when tumor had mixed SI area, SI from the majority ( at least 60% ) of the solid part of mass was scored by chosen from single slice axial view with greatest dimension of the tumor.After independently reviews were done, discrepancy was rescored by consensus between two reviewers. 

*ADC value measurement*

ADC value of glioma in single slice axial view was measured by one neuroradiologist, after choosing the location for measurement by consensus of two reviewers. For ADC value measurement, free hand circular regions of interest (ROI) was done to cover the whole solid portion in single axial slice with greatest dimension of tumor, The ROI varied from 22.67 mm^2^ to 1675.28mm^2^ in areas and were adjusted to include only regions of the solid tumor based on the other nonenhanced and enhanced MR images. Areas of necrosis, cyst, hemorrhage, edema, and calcification were avoided. 

*Statistical analysis*

All eligible patients were classified into two groups by the WHO criteria for glioma grading: low grade (WHO grade I, II) and high grade (WHO grade III, IV). The data of visual scale and ADC values were compared with tumor groups of the low and high grade glioma. Visual scale comparison between two groups was analyzed by using chi-squared test. ADC value comparison was analyzed by using Mann-Whitney U test. P-value less than 0.05 indicated a statistically significant difference. Shapiro-Wilk test was used to check normality assumption for all parameters in all groups (P<0.05 indicated non-normal distribution). Quantitative value in normal distribution was presented as mean ± standard deviation, while those in non-normal distribution was presented as median (interquartile range). Analysis of receiver operating characteristic (ROC) curve was performed to determine optimum threshold for tumor grading and also to calculate the sensitivity, specificity and accuracy for identifying high grade. The best cutoff point of ADC value to identify high grade glioma was determined by the highest sensitivity and specificity. Finally, the comparison was done between the visual scale and ADC values with the WHO grading glioma groups.For the statistical analysis of the obtained data, software package STATA version10 (Stata Corp2007, Stata Statistical Software: Release10, College Station, TX:Stata Corp, LP) was used.

## Results

*Demographic data*

During the study period, there were 56 patients with histopathologically proven gliomas. Of those, 38 patients (67.85%) had the preoperative DWI and 20 patients (52.63%) had the high grade glioma including anaplastic oliogodendroglioma (n =1), ependymoma WHO3 (n = 2), gliomatosis cerebri (n = 1), anaplastric astrocytoma (n=9), Glioblastoma (n=7). For the low grade glioma 18 patients , the histopathology findings were pilocytic astrocytoma (n = 3), diffuse astrocytoma (n=10), oligodendroglioma (n=4), and pilomyxoid astrocytoma (n=1). There were no significant different in terms of age and sex between the low and high grade group (Table 1). The median age of all patients was 38.50 years (range 0.91-72) with somewhat higher proportion of male sex (55.26%).

*Qualitative Assessment*

High grade gliomas had visual scale 4 and 5 which exhibit hyperintensity on DWI. No low grade glioma had visual scale 5. Visual scale 2 or 3 was found only in low grade. However visual scale 4 could be found in both low and high grade. No cases had a visual scale of 1. 9 Figures 4-8) The variety of visual scale among different group was shown on Table 2. Chi-squared test was used to compare visual scale between two groups of glioma due to categorical variable which showed overall statistical significance with P = 0.002. If we use the cutoff point of visual scale 5, the sensitivity, specificity, PPV NPV and accuracy were 50%, 100%, 100% , 64.3%,73.68% respectively. 

*Quantitative Assessment*

The high grade group had significantly lower ADC value than the low grade group (969.12 vs 1,470.02 x10^-6^ mm^2^/s) as shown in Table 2. The best ADC cut off point was 1,119.48 x10^-6^mm^2^/s for tumor grading, resulting in the sensitivity, specificity, PPV, NPV and accuracy were 88.90%, 90 %, 90%, 88.9%, 89.47% respectively.

*Comparison of visual scale and ADC to the WHO grading*

**Figure 1:**
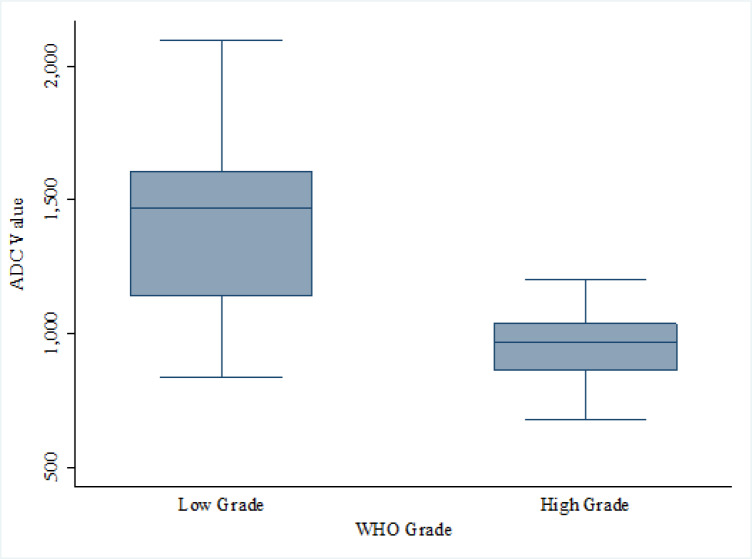
Box Plot of ADC Value between Low Grade and High Grade Glioma. Note, ADC value are expresses in X 10^-6^ mm^2^/s

**Figure 2:**
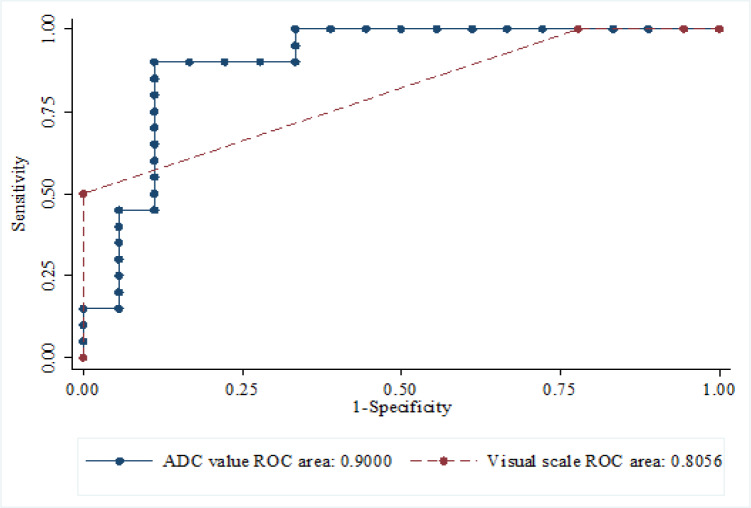
Comparison Test Accuracy between ADC Value and Visual Scale by ROC Analyses (Gold Standard = WHO Grade) (P = 0.163)

The ADC value had higher area under the ROC curve than the visual scale (90.00 vs 80.56) as shown in Figure 1. However, there was no statistical significant difference between both areas under the ROC (p value 0.186), as shown in Figure 2. 

**Table 1:** Description of Mean Age, Difference of Sex and Patient Number between Low Grade and High Grade Glioma

Demographic	Total	WHO Grade	
	(n=38)	Low (n=18)	High (n=20)
Sex			
Female	17 (44.74)	8 (44.44)	9 (45.00)
Male	21 (55.26)	10 (55.56)	11 (55.00)
Age			
Mean (SD)	36.68 (22.14)	31.28 (22.60)	41.55 (21.10)
Median (min-max)	38.5 (0.91 - 72)	29 (0.91 - 72)	50 (1 - 71)

**Table 2:** The Variety of Visual Scale among Different Groups

Visual scale	WHO Grade		P-value	
	Low (n=18)	High (n=20)		
1	0 (0.00)	0 (0.00)	NA	0.002
2	1 (5.56)	0 (0.00)	0.474	
3	3 (16.67)	0 (0.00)	0.097	
4	14 (77.78)	10 (50.00)	0.101	
5	0 (0.00)	10 (50.00)	<0.001	

Note, In this series, there were no tumors with a visual scale of 1. Number in parentheses are percentage of tumor number

**Table 3:** Comparison of ADC between Two Groups of Gliomas

	WHO Grade		P-value
	Low (n=18)	High (n=20)	
ADC value: Median (range)	1470.02 (837.83 – 2099.56)	969.12 (678.66 – 1202.70)	<0.001
Mean (SD)	1396.95 (312.73)	948.31 (148.92)	

Note, ADC value are expresses in X 10^-6^ mm^2^/s.

**Figure 3:**
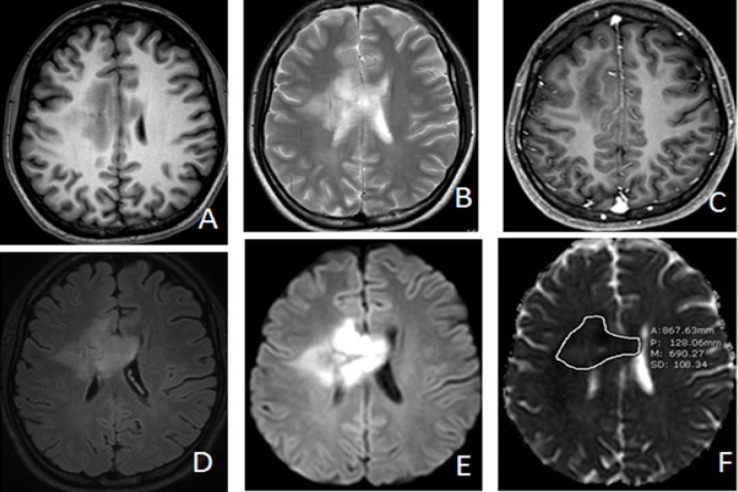
Anaplastic Astrocytoma (WHO Grade III) in 19 Year-Old-Woman. Axial T1W (A), T2W(B), Post contrast T1WFS(C), FLAIR (D), DWI(E) and ADC map (F) images representing case that showed marked hyperintense solid part of the tumor with visual scale 5 on DWI (E) and ADC value measurement = 690.27 x 10^-6^ mm^2^/s on ADC map(F) which is below the cutoff value

**Figure 4:**
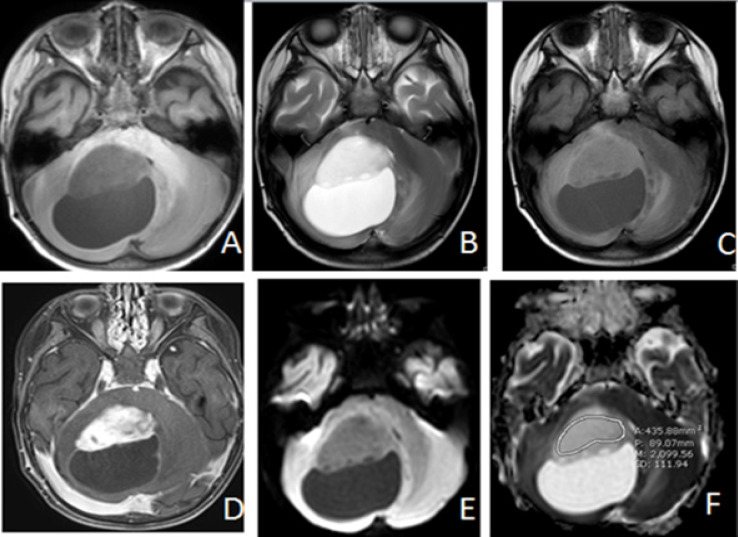
Pilocytic Astrocytoma (WHO Grade I) in 2 Year-Old-Boy. Axial T1W(A), T2W(B), FLAIR(C), Post contrast T1WFS(D), DWI (E) and ADC map (F) images representing case that showed hypointense solid part of the tumor with visual scale 2 on DWI(E) and ADC value measurement = 2099.56 X 10^-6^ mm^2^/s on ADC map (F) which is much higher than the cutoff value

## Discussion

Distinguishing low grade from high grade gliomas on the basis of conventional MR imaging findings can be challenging since high grade and low grade gliomas can have overlapping features on MR imaging (Sugahara et al., 1999; Okamoto et al., 2000; Kono et al., 2001). DWI provides additional unique information derived from microscopic motion of the water molecule, which cannot be obtained with conventional MRI. DWI is widely used in clinical practice and is routinely performed on brain MRI protocols. 

Visual assessment is an easy and rapid method for grading of tumors which does not require a specialized postprocessing workstation (Kono et al., 2001; Seo et al., 2008).The simplest approach is to record whether restricted diffusion is visually present (bright area on the b 1000 trace image and a corresponding dark area on ADC map) (Kan et al., 2006). This can be done even for older or outside studies in which only filmed or scanned images are available. Okomo et al., (2000), reported mainly iso to hypointensity compared to gray matter in low grade and moderated to markedly hyperintensity in nonenhanced, enhanced high grade gliomas respectively. Sugahara et al., (1999), reported low grade gliomas exhibit iso to mild hyperintensity compared with gray matter on DWI, but more hyperintensity in glioblastoma. The present study found the majority of low grade gliomas having mild hyperintensity or visual scale 4 and less hypo to isointensity (visual scale 2 to 3). These results differ from those of previous reports (Sugahara et al., 1999; Okamoto et al., 2000) and may be explained by using different reference tissue between gray matter in previous studies and subcortical white matter in our study. Our study found high grade glioma had hyperintensity on DWI with visual scale 4 and 5 equally which agree with previous studies (Sugahara et al., 1999; Okamoto et al., 2000; Seo et al., 2008). Notably, none of the low-grade tumors in the present study demonstrated marked hyperintensity or visual scale 5 and confirmed that marked DWI hyperintensity or visual score 5 can effectively distinguish high-grade from low-grade tumors. The increased cellularity, decreased extracellular space, and high nuclear-to cytoplasmic ratio are currently believed to be the factors responsible for microscopic water movement restriction in tissue of high-grade tumors resulting in a higher DWI signal intensity than in low-grade tumors. (Castillo et al., 2001; Stadnik et al., 2001; Lam et al., 2002; Higano et al., 2006; Van et al., 2006; Rollin et al., 2006; Lee et al., 2008; Murakami et al., 2009; Chen et al., 2010). Using the cutoff of visual scale=5 to predict high grade glioma, our results demonstrated sensitivity=50 %, specificity=100%, PPV=100%, NPV=64.3% and accuracy =3.68 %. Our study compares favorably to a study Seo et al., 2008 which compared different b-value diffusion MRI with slightly larger sample size and reported a diagnostic accuracy = 70%,. sensitivity = 70% and specificity = 76.9%, PPV = 94.4% and NPV = 40%. Shoaib et al., (2019) compared diagnostic performance between diffusion MRI and perfusion MRI and reported a sensitivity = 69.57% , specificity = 75%, PPV= 88.8%, NPV =46.15% diffusion MRI. 

Quantitative assessment of ADC value is a useful and reproducible parameter that has been widely used to evaluate tumor grade or cellularity (Sugahara et al., 1999). The reason we used a single large freehand ROI to cover solid part of the tumor was to assist in Correlating specimen histopathology and ADC. In addition, a previous study by Han et al., (2017) had found no difference in measured ADC value between multiple small ROI or single freehand ROI methods. We used a single axial slice showing the largest tumor dimension since whole-volume histogram analysis did not yield better results than single-slice method for ADC (Wang et al., 2018). Kono et al., (2001), found ADC may predict the degree of malignancy of astrocytic tumors, although there is some overlap between ADC of grade II astrocytoma and glioblastoma. But Lam et al., (2002) and Rollin et al., (2006), found the differentiation between high- and low-grade gliomas was not possible using diffusion-weighted images and ADC value alone. Some previous studies found the signal characteristics on DWI and ADC maps appeared to be strongly correlated to grade in pediatric brain tumors and neuroepithelial tumors (Kan et al., 2006; Jaremko et al., 2010; Chen et al., 2010). We found the ADC values in high grade tumors to be significantly lower compared to low grade gliomas. Higher ADC values in low grade tumors may reflect an increase the water content of interstitial spaces, less cellularity and low nuclear to cytoplasmic ratio. We found very high ADC value in pilocytic and pilomyxoid astrocytoma because of similar loose cellular pattern. When 1,119.48 x 10^-6^mm^2^/s was used as the optimal cutoff mean ADC value, a combination of sensitivity (90%) and specificity (88.9%) was achieved to distinguish between low and high grade glioma which was the same range of cutoff value in prior studies(Lee et al., 2008; Cihangiroglu et al., 2017). Our cutoff value is higher than some previous studies which was 900-1,000 x10^-6^ mm^2^/s which was due to using minimum ADC values instead of mean ADC values (Higano et al., 2006; Murakami et al., 2009; Wang et al., 2018). 

For comparison between qualitative and quantitative measurement with grading of gliomas, we found AUC of visual scale was 0.81 and AUC of ADC measurement in grading gliomas was 0.9 which indicated a good level for diagnostic efficacy. Although there was no statistically significant difference between the AUC between the qualitative and quantitative methods, the lower sensitivity, NPV and accuracy of the visual assessment compared to the quantitative assessment suggest that visual scale may not replace ADC measurement. However, the qualitative evaluation of marked hyperintensity on DWI hyperintensity is relatively easy and more efficient yielding similar results. 

There were several limitations in our study. The main limitation is relatively small sample size. However, the power of this study was 98.83% based on our sample size of 38. Second, this was a retrospective study, and our promising early results need to be confirmed with larger prospective trials. 

In conclusion, both visual scale and ADC value were capable of differentiating between low and high grade gliomas. Although ADC measurement is a more accurate method of glioma grading, our results indicate that visual scale can also be used to assess histologic grading and our initial diagnostic performance results warrant confirmation with larger prospective studies. 
